# Fingolimod (FTY720) Preserves High Energy Phosphates and Improves Cardiac Function in Heterotopic Heart Transplantation Model

**DOI:** 10.3390/ijms21186548

**Published:** 2020-09-08

**Authors:** Naseer Ahmed, Javeria Farooq, Soban Sadiq, Sultan Ayoub Meo, Azam Jan, Faisal H. Cheema, Giuseppe Faggian, Alessio Rungatscher

**Affiliations:** 1 Department of Biological and Biomedical Sciences, The Aga Khan University, Karachi 74800, Pakistan; javeria.farooq@aku.edu; 2 Department of Surgery, Cardiac Surgery Division, University of Verona Medical School, 37129 Verona, Italy; giuseppe.faggian@univr.it; 3 Faculty of Medicine, Imperial College London, London SW72AZ, UK; s.sadiq@imperial.ac.uk; 4 Department of Physiology, College of Medicine, King Saud University, Riyadh 11461, Saudi Arabia; smeo@ksu.edu.sa; 5 Department of Cardiac Surgery, Rehman Medical Institute, Peshawar 25000, Pakistan; azam.jan@rmi.edu.pk; 6 Department of Biomedical Sciences, University of Houston College of Medicine, Houston, TX 77004, USA; faisal.cheema@me.com

**Keywords:** fingolimod (FTY720), cardiac function, coronary blood flow, high energy phosphates, poly (ADP ribose) polymerase

## Abstract

During heart transplantation, donor heart leads to reduced oxygen supply resulting in low level of high energy phosphate (HEP) reserves in cardiomyocyte. Lower HEP is one of the underlying reasons of cell death due to ischemia. In this study we investigated the role of Fingolimod (FTY720) in heart transplantation ischemia. Eight groups of Sprague-Dawley rats (*n* = 5 for each subgroup) were made, A1 and C1 were given FTY720 1 mg/kg while B1 and D1 were given normal saline. The hearts were implanted into another set of similar rats after preservation period of 1 h at 4–8 °C. Significantly higher Left ventricular systolic pressure (LVSP), dP/dT maximum (*p* < 0.05), dP/dT minimum (*p* < 0.05) were recorded in the FTY720 treated group after 24 h of reperfusion while after 1 h of reperfusion, there were no significant differences in LVSP, maximum and negative dP/dT, and Left ventricular end diastolic pressure (LVEDP) between the control and the FTY720-treated transplant groups. Coronary blood flow (CBF) was enhanced (*p* < 0.05) in the FTY720 treated group after 1 and 24 h. ATP *p* < 0.001, *p* < 0.05 at 1 and 24 h, ADP *p* < 0.001, *p* > 0.05 at 1 and 24 h, and phosphocreatine *p* < 0.05, *p* > 0.05 at 1 and 24 h were better preserved by FTY720 treatment as compared to control group. The study concluded that pretreatment of grafted hearts with FTY720 improved hemodynamics, CBF, high energy phosphate reserves, reduces the peroxynitrite level and poly (ADP ribose) polymerase (PARP) inhibition that prevents ischemia-reperfusion injury.

## 1. Introduction

Heart failure (HF) is the leading complication of many cardiovascular diseases [1,2]. The ultimate treatment for advanced heart failure is heart transplantation [3]. During ischemia, compromised blood supply results in decrease supply of oxygen and low level of glucose that leads to the utilization of glycogen and initiation of anaerobic glycolysis. After some time, glycolysis is inhibited by its products which limits the anaerobic ATP production. Levels of ATP, glycogen, and creatinine phosphate remain closely related in an ischemic myocardium [4]. Depletion of high energy phosphates is linked with harmful injury in acutely ischemic myocardium. It is observed that more than 90% of the ATP gets depleted after 40 min of ischemia and is related with major modifications in cell structure [5]. Nitric oxide (NO) plays a major role in vascular homeostasis and regulating apoptosis. NO is known as toxic and reactive because of its cytotoxic metabolite peroxynitrite (ONOO^−^), which is formed due to reaction between NO and superoxide anion. Presence of peroxynitrite exacerbates myocardial damage during ischemic reperfusion injury. Peroxynitrite stimulates nuclear enzyme PARP, that can lead to impaired cardiovascular functions and inflammatory disorders [6].

Fingolimod (FTY720) is novel, less toxic, and has a better immunosuppressive effect on transplanted organ [7]. FTY720 regulates S1P_1_ activities that are essential for lymphocyte migration and trafficking [8]. FTY720 is an effective anti-inflammatory and antioxidant agent, it inhibits the production of oxygen free radicals which reduce myocardial fibrosis and decreases the number of cardiomyocytes death [9,10,11]. From previous experimental studies it is evident that PARP (Poly(ADP-ribose) Polymerase) plays an active role in modulating pathophysiological condition of myocardial injury [12]. It has been found that FTY720 inhibits PARP cleavage and cytoprotection [13].

In this study we evaluated the pretreatment effects of FTY720 on heterotopic heart transplantation model. Our hypothesis also included that FTY720 might play role in cardioprotection with inhibition of PARP-1 pathway in our model. The purpose of the present study was to explore the effects of FTY720 on hemodynamic parameters, coronary blood flow, reservation of high energy phosphates, and peroxynitrite expression.

## 2. Results

### 2.1. Early Reperfusion (Groups A and B)

Baseline parameters in both groups were measured as mentioned in (Table 1). The hemodynamic points and CBF were calculated after 60 min of reperfusion in groups A and B. Systolic functional activity was found to be markedly better in the FTY720 group as compared to control. LVSP and dP/dT max were significantly improved (*p* < 0.05) in the FTY720 treated group. Systolic cardiac function graphs showed a major shift in the FTY720 treated group in comparison to the control-saline treated group.

**Figure 1:**
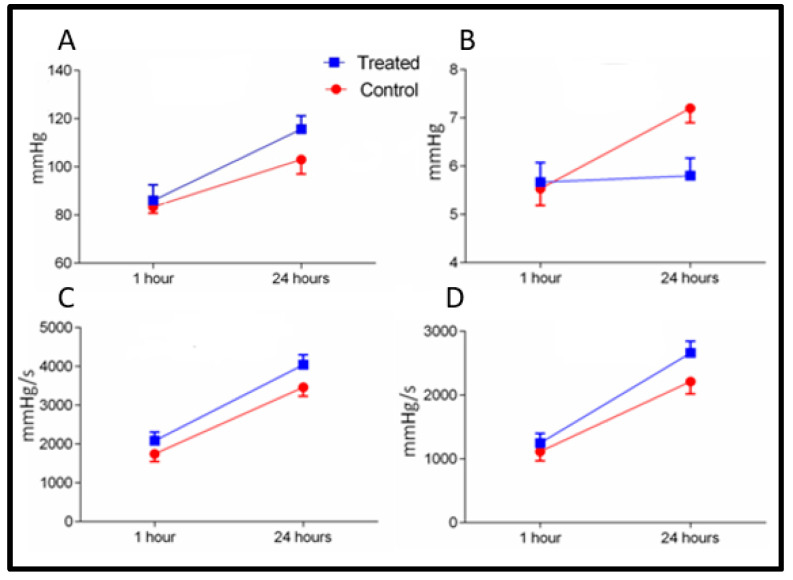
Hemodynamic parameters at 1 and 24 h of myocardial reperfusion in FTY720 and Control-saline treated groups. (**A**) Effect of FTY720 on LVSP, (**B**) LVEDP, (**C**) LV dP/dT max, (**D**) LV dP/dT min. Values are expressed as the means ± SD. dP/dT max: the rate of maximum positive left ventricular pressure development; dP/dT min, the rate of maximum negative left ventricular pressure development LVSP, left ventricular systolic pressure; LVEDP, left ventricular end-diastolic pressure.

**Figure 2:**
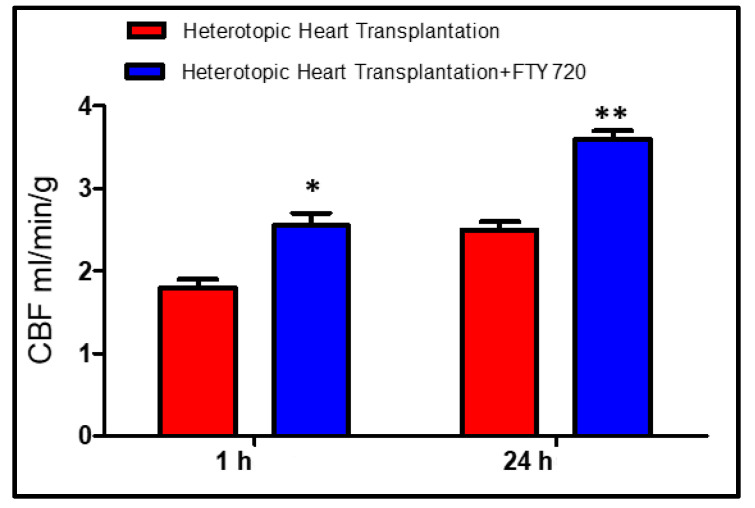
Coronary blood flow (CBF) measurements. Group A, B: early reperfusion at 1 h, Group C, D: late reperfusion at 24 h of reperfusion. * *p* < 0.05, ** *p* < 0.001.

Minimum dP/dT was significantly higher (*p* < 0.001) in the FTY720 group leading to myocardial relaxation. LVEDP did not change between the groups (Figure 1). The diastolic function curves (end-diastolic pressure–volume relationships) were similar in all groups. CBF was significantly increased (*p* < 0.05) in the FTY720 group as compared to control group after 1 h (Figure 2).

### 2.2. Late Reperfusion (Groups C and D)

After 24 h of reperfusion, there was significant difference in readings of LVSP, maximum and minimum dP/dT, and LVEDP between the control and the FTY720-treated transplant groups (Figure 1). The CBF values were improved (*p* < 0.001) in the FTY720 group in comparison to control after 24 h of reperfusion (Figure 2).

### 2.3. High-Energy Phosphates

**Figure 3:**
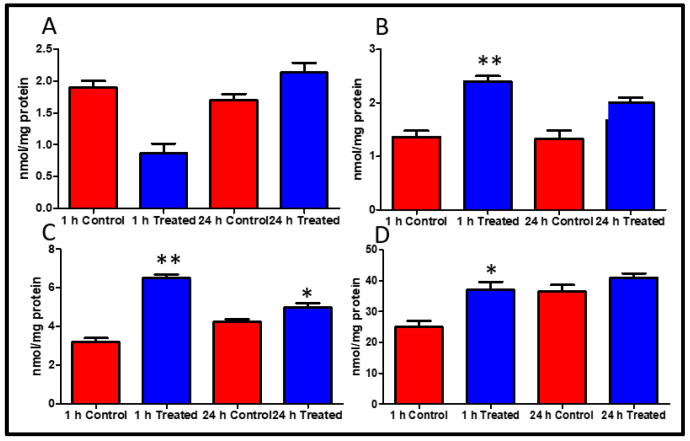
(**A**) AMP, (**B**) ADP, (**C**) ATP, and (**D**) changes in phosphocreatine levels in left ventricular tissue of the heart in FTY720treated groups and control-saline treated group at 1 and 24 h of reperfusion. AMP: adenosine monophosphate. ADP: adenosine diphosphate. ATP: adenosine triphosphate. Data presented as a mean ± SD. *p*-value < 0.05 considered as significant. (∗ *p* < 0.05 and ∗∗ *p* < 0.001, treated vs. control).

Myocardial high energy phosphate particularly ATP and ADP content were better conserved by FTY720 treatment during heart transplantation. The levels of AMP at 1 and 24 h were not considerably improved in the treated group *p* = 0.2, *p* = 0.06 (Figure 3A). The level of ADP and ATP were significantly improved at 1 and 24 h in the FTY720 treated group (Figure 3B,C). Phosphocreatine (PCr), the buffering energy source for ATP in circumstances of energy requirement, was significantly increased in the FTY720 treated group of animals as compared to control group myocardial tissue at 1 h while no difference was detected at 24 h (*p* < 0.05, *p* > 0.05, 1 and 24 h) (Figure 3D).

### 2.4. Peroxynitrite Expression

**Figure 4:**
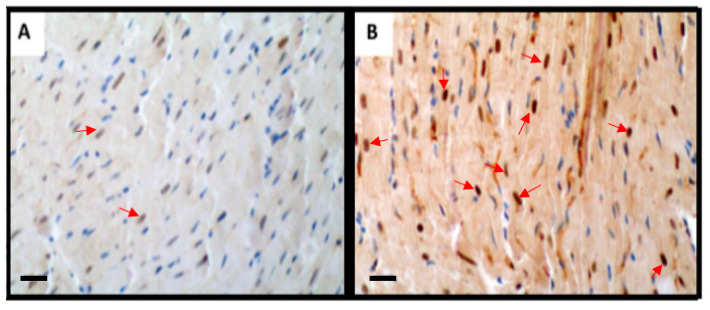
Myocardial nitrotyrosine staining, magnification 20×, scale bar 100 µm, blue color representing nuclei, transplanted heart tissue was collected after 24 h of reperfusion for nitrotyrosine localization by immunohistochemistry. (**A**) FTY720-treated group, (**B**) control-saline treated group. Red arrows illustrating decreased expression of peroxynitrite in treated group as compared to control.

Excessive nitric oxide (NO) formation from nitric oxide synthase (NOS) and the reaction between NO and superoxide resulted in peroxynitrite release which plays a role in activation of apoptotic signaling pathways leading to apoptotic cell death. To determine whether FTY720 can attenuate myocardial nitrative stress peroxynitrite expression was evaluated. As demonstrated in Figure 4, FTY720 markedly downregulated peroxynitrite expression, that indicates decreased production of nitrotyrosine.

### 2.5. Caspase 3 and PARP-1 Inhibition

**Figure 5:**
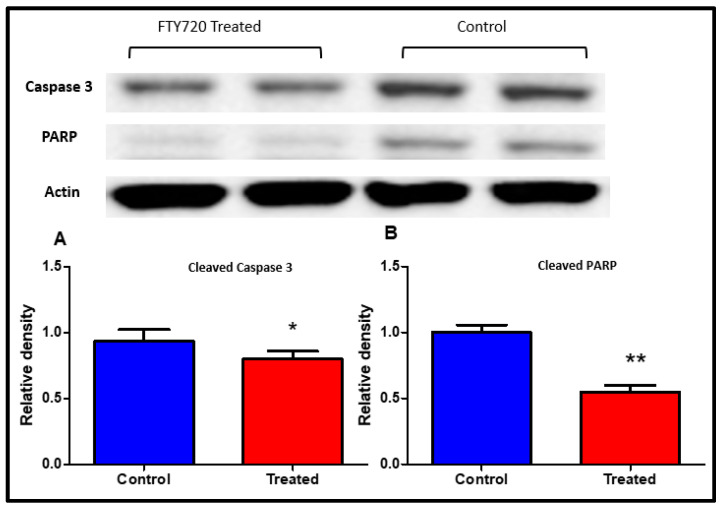
Cleaved caspase-3 and PARP protein expressions in myocardial tissue analyzed using Western blot. (**A**,**B**) Relative densities of cleaved caspase-3 and PARP protein levels. Data presented as a mean ± SD. *p*-value < 0.05 considered as significant. (∗ *p* < 0.05 and ∗∗ *p* < 0.001, treated vs. control).

Apoptosis is the major concern during ischemia reperfusion. In this study, the effect of FTY720 on Caspase 3 and Poly (ADP Ribose) polymerase inhibition in myocardial tissue following heterotopic heart transplantation was studied. As shown in Figure 5, the control group induced more activation of apoptosis, as observed by enhanced expression of cleaved caspase-3 and cleaved PARP as compared to FTY720 treated group. FTY720 attenuated cleavage of caspase 3 and PARP in the transplantation model.

## 3. Discussion

The results from this study suggested that the preconditioning of heart with FTY720 may exert a cardioprotective effect. In previous studies, we investigated the effect of FTY720 in various ischemia reperfusion [10,14,15,16,17] and transplantation models [16]. We found a cardioprotective effect of this drug on sudden cardiac arrest and cardiopulmonary bypass. Additionally, we found this drug to have a particular effect on myocardial fibrosis in a transplantation model [16]. In this current study, we reported, for the first time, an effect of FTY720 on Poly (ADP Ribose) polymerase signaling, HEP preservation, and coronary blood flow in heterotopic heart transplantation. We observed that the hearts treated with FTY720 before transplantation had better hemodynamics, coronary blood flow, and high energy phosphate reserves as compared to the saline treated group. Furthermore, coronary blood follow was better at the first hour after implantation. This may be responsible for the better high energy phosphate store observed in this group. As discussed earlier, ischemia is responsible for shift in metabolism and anaerobic glycolysis with reduced ATP production. Even anaerobic glycolysis is inhibited by its own products within a certain time of loss of blood supply leading to loss of ATP production [4]. Our study has shown that FTY720 treated hearts had better ATP and phosphocreatine reserves. FTY720 may be involved in delaying the shift in metabolism and production of a higher amount of ATP, thus reserving phosphocreatine as well. NAD^+^/NADH ratio is also an indicator of energy reserve. NAD+ is released by mitochondrial permeability transition pore opening in myocytes. Loss of NAD+ from mitochondria results in PARP-1 activation [18]. The cardioprotective effect of sphingosine 1-phosphate from ischemic reperfusion injury may be exhibited by interfering with the PARP activation pathway [19]. PARP is involved in cell death when there is higher oxidative stress [20]. FTY720 is also involved in reducing oxidative stress thus stabilizing mitochondrial membrane. Myocardial ischemia-reperfusion also produces nitric oxide synthase, that release nitric oxide which produce toxic metabolite peroxynitrite that also behaves as ROS leading to necrosis and apoptosis. Peroxynitrite formation after ischemia-reperfusion causes overstimulation of PARP-1 leading to cell death by NAD^+^/ATP depletion [21]. Fingolimod treatment partially attenuates peroxynitrite expression in transplanted myocardium.

In literature, it was demonstrated that purine triphosphate levels decreased while purine monophosphate, base, and hypoxanthine levels were increased in heart with reperfusion after 30 min who underwent coronary bypass grafting [22]. Inhibition of poly (ADP ribose) polymerase prevents endothelial and myocardial injury during reperfusion [23]. However, it is also observed that ischemia aggravates apoptosis. The quantity of apoptotic cells relates to the high-energy phosphate depletion in a direct approach [24]. Scientists have focused on investigating the role of different physiological and biochemical conditions to preserve high energy phosphate stores ultimately reducing ischemic reperfusion injury. In a study on pig hearts, normothermic ischemia in hearts isolated for transplantation had lower ATP as compared to those exposed to immediate reperfusion [25]. However, studies on pigs have shown no effect of cold static storage on high energy phosphate content in hearts from brain-dead animals [26]. Another study has reported that exogenous phosphocreatine injection given along with CPR in rats, reduces cardiomyocytic apoptosis and I/R injury but at a very high dose [27].

**Figure 6:**
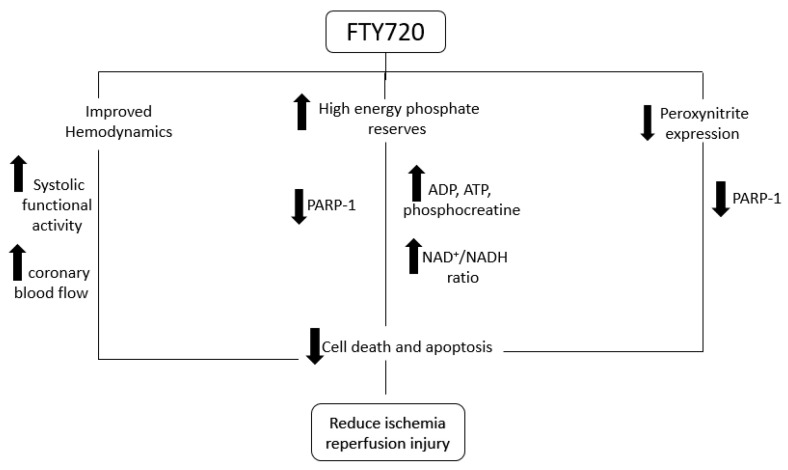
Proposed mechanism of action of FTY720 in ischemia reperfusion injury (upward arrows showing increase and downward mean decreasing).

Figure 6 shows the proposed mechanism of action of FTY720 according to our study findings. Thus, our study has shown that pretreatment with FTY720 is an effective alternate for preserving high energy phosphate content of cardiomyocytes (Figure 3). Like previous studies [28,29,30], this study also reported that FTY720 is involved in inhibiting PARP induced injury which may be involved in limiting cell death through apoptosis (Figure 5). Our study has shown better hemodynamics in the treatment group which may be due to reduced apoptosis in cardiomyocytes. These findings suggest that fingolimod can be efficiently used as a pharmacological preconditioning agent to improve myocardial salvage caused by PARP-1 inhibition and activation by reducing peroxynitrite expression (Figure 4). FTY720 is reported for its role in minimizing endothelial dysfunction [27]. This is also supported by our finding of better coronary blood flow in the FTY720 treated group. FTY720 may be used as a preconditioning agent for heart and possibly other organ transplantation. It can potentially be used before coronary artery bypass grafting [17], in myocardial infarction, pulmonary embolism, stroke, and many similar conditions. Human studies are required to be conducted in this regard to explore beneficial effects, therapeutic dose, and side effects of FTY720 in recommended conditions.

In our study, the maximum reperfusion time was 24 h, extended time may provide more strength and evidence in similar study. However, it was challenging due to inclusion of difficult surgical procedures. Fingolimod is a safe and well tolerated drug but certainly has limitations. Although some studies have suggested atrioventricular block (AVB), the incidence of Mobitz Type 1 is extremely low whereas higher degree heart blocks such as Mobitz Type 2 or third-degree AVB have been rarely associated with FTY720. Conduction abnormalities, if any, have been observed to regress over time. Furthermore, this limitation can be addressed with careful titration of therapeutic dosing in order to bring about the use of FTY720 in the setting of heart transplantation with an intent for this pharmaceutical agent’s translation into the clinical realm. Further studies are required to implement the protective effect of fingolimod into clinical practice.

## 4. Materials and Methods

Sprague-Dawley rats (300–350 g) were taken from Harlan Laboratories (Udine, Italy). Two rats were placed per cage and they received the standard diet (rat chow) and had free access to water. They were kept in light/dark cycle of 12 h to ensure circadian rhythm and were maintained at a temperature of 21 °C. The study protocol was approved by The Ethical Committees of the University of Verona and National Animal Welfare Committee (BB-CCH#13371, 16 March 2016) and Aga Khan University (ECACU# 83-ECACU-BBS-18, approved on 15 November 2019).

In the study, rats were divided into eight transplant groups with five rats in each group: A1, B1, C1, D1, A2, B2, C2, and D2 (Figure 1). Groups A1, B1, C1, and D1 were donor rats while A2, B2, C2, and D2 served as recipients for these abdominal heart transplantation procedures. Two of the donor groups A1 and C1 were given a single intravenous dose of 1 mg/kg of Fingolimod (FTY720) and thus constituted the treated groups. Whereas, the other two donor groups B1 and D1 were administered with normal saline intravenously and served as control groups. Slow injection of both saline and FTY720 (1 mg/kg) were administered 15 min before introducing the aortic cross clamp. Recipient rats were anesthetized with 5% isoflurane in 50% O_2_. Abdominal incision was made and sub-renal aortic and inferior vena cava (IVC) were exposed and isolated. Two vascular clamps were positioned cranially and caudally to the site of transplanted heart implantation. Saline solution and heparin were used to avoid thrombus formation. Concurrently, the donor rat was also prepared. Anesthesia was introduced by intraperitoneal administration of sodium thiopental (Pentotal^®^) 60 mg/kg. The rat was intubated orotracheally with an atraumatic tube consisting of a 16 G venous cannula and was mechanically ventilated (Harvard Model 687; Harvard Apparatus, Holliston, MA, USA). Tidal volume was set to 10 mL/kg and respiratory rate 60 breaths/min with an air–oxygen mixture/FiO_2_ = 0.5. Anesthesia was maintained with isoflurane (2%) during the whole procedure. Median sternotomy was carried out to expose donor hearts. The right superior vena cava (SVC) was tied with Ticron 3.0 suture, ascending aorta and pulmonary artery were resected at 3–4 mm from their origin with an angled scissor. Cold S. Thomas cardioplegic solution was injected to stop the heartbeat, and after that IVC was tied and closed with Ticron 3.0 suture. The heart was then lifted up and pulmonary veins and left SVC were tied and closed with a single ligature. The heart was then harvested and immediately dipped in a cold cardioplegia bath and another 10 mL of S. Thomas solution was injected into the heart through ascending aorta to perfuse it similar to the perfusion technique explained in our previous work [16].

**Figure 7:**
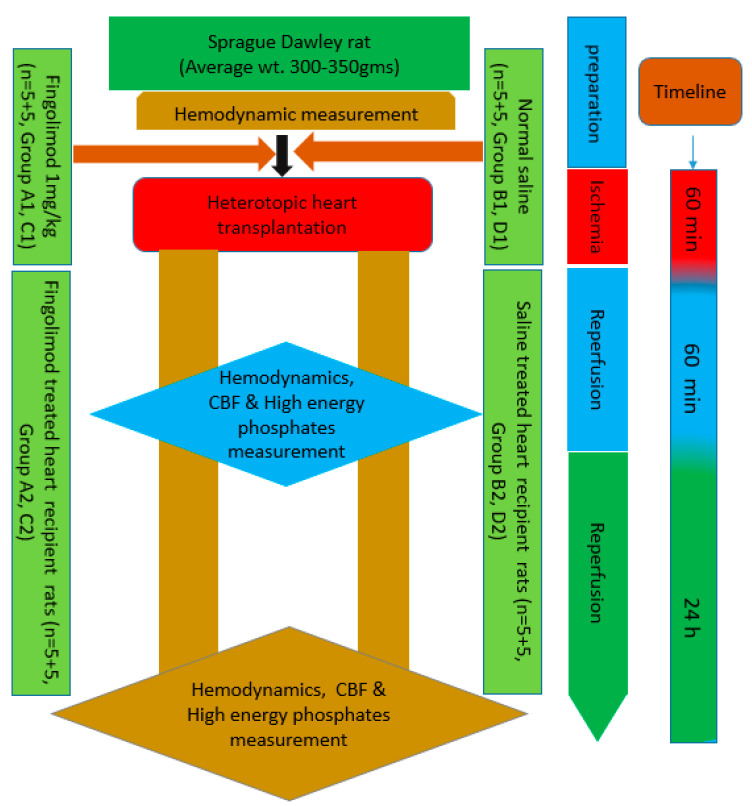
Experimental design.

The explanted heart was anastomosed with abdominal aorta of the recipient rat and positioned in order to avoid twisting. The first anastomosis was performed between the abdominal aorta of the recipient rat and the ascending aorta of the donor heart using polypropylene 8.0 continuous suture. After completing this anastomosis, the anastomosis between IVC of the recipient rat and the pulmonary artery of the donor heart was performed. The heart was topically cooled by irrigating intermittently with saline solution at 4 °C during the transplant procedure. Time for the execution of the two anastomoses was between 35 and 40 min, but total ischemic time was 60 min in all experiments that was controlled by removal of aortic clamps. If no stenosis or bleeding was evident, the implanted heart spontaneously starts beating. Warm saline solution was poured into the abdominal cavity and the abdomen was closed through continuous suture of the muscle wall and of the skin with a Polypropylene 4.0 suture. Sedation was suspended and animals were given time to recover from the anesthesia [16]. Systolic and diastolic function and CBF readings were measured after 1 and 24 h. Whereas Groups A2 (recipient rats in the treated group) and B2 (recipient rats in the control group) were only survived for 1 h while Groups C2 (recipient rats in the treated group) and D2 (recipient rats in the control group) were survived for 24 h. During those 24 h, animals of both groups (C2 and D2) were fed with the standard diet and normal water was given for drinking. After 24 h the rats in groups C2 and D2 were again given anesthesia and the abdominal cavity was opened again to harvest the transplanted hearts for further analysis [16,31]. Experimental design is demonstrated in Figure 7.

### 4.1. Functional Measurements of the Graft

The measurements of left ventricular systolic pressure (LVSP), left ventricular end-diastolic pressure (LVEDP), pressure development rate (dP/dT) maximum and minimum were done by using midline of the neck. The right carotid artery was isolated, after making a small incision, the catheter was inserted (model SPR 838, Millar Instruments, Houston, TX, USA) into the carotid artery. The catheter was then attached to the transducer and to Power-Lab (AD Instruments, Colorado Springs, CO, USA). Conductance was introduced into the carotid artery and advanced to the left ventricle, the correct position was reached by following the trend of the pressure The signal was recorded continuously with a sampling rate of 1000/s thus being able to monitor the hemodynamic changes during the whole duration of the intervention [14,15,17]. After cardiac arrest the superior and inferior caval veins and the pulmonary veins were tied with a suture and the heart was excised with the aortic arch for measurement of the coronary blood flow (CBF). CBF of the graft was calculated using an ultrasonic flow meter (Transonic Systems Inc., Ithaca, NY, USA) which was placed on ascending aorta segment of the donor heart. Thus, the ultrasonic flow probe measures the blood flow retrogradely in the donor aortic segment from the aorta (of recipient rat) toward coronary vessels of transplanted heart, which is equal to CBF in our heterotopic transplantation model.

### 4.2. High-Energy Phosphates Measurement

The level of adenosine monophosphate (AMP), adenosine diphosphate (ADP), adenosine triphosphate (ATP), and phosphocreatine (PCr) were measured in myocardial tissue. For collecting tissue samples from beating hearts, clamps preloaded with liquid nitrogen precooled were used. The sample of myocardial tissue was kept at −80 °C. Tissue samples were deposited in liquid nitrogen until further processing. Then, 50–100 mg of frozen tissue was deproteinized using 500 µL of 0.4 mol/L perchloric acid (Braun, Melsungen, Germany). Centrifugation of 150 µL of acid extract was done at 12,000× *g*, then 2 mol/L potassium carbonate was added for neutralization at 4 °C. Again, centrifuged and the supernatant was stored at −28 °C till further procedures [17]. Hypersil ODS column (5 μm, 250 mm long × 4 mm ID) with an AS-100 HPLC automatic sampling system (Bio-Rad, Hercules, CA, USA), was used for chromatographic separation. Detector signals (absorbance at 214 nm for PCr and 254 nm for adenine dinucleotide) were recorded with an AGC personal computer. For data requisition and analysis system Gold (Beckman Coulter Inc., Brea, CA, USA) was used as controller. The extract pellets were dissolved in 1 mL of 0.1 mol/L sodium hydroxide and diluted with ratio of 1:10 in saline for protein determination using BCA assay kit (Beyotime Institute of Biotechnology, Jiangsu, China) [32].

### 4.3. Measurement of Peroxynitrite Expression

The measurement of nitrotyrosine was used for assessment of plasma peroxynitrite concentrations. Immunohistochemical studies demonstrated that the ONOO (-)-mediated nitration product nitrotyrosine was formed by measuring the nitrosylation of cardiac proteins using an antibody against nitrotyrosine. Myocardial tissue sections with thicknesses of 3 microns were taken and placed on polarized slides and heated for 1 h at 60 °C. The sections were then placed in xylene and ethanol for 20 min for rehydration. After addition of 0.01 M citrate buffer at pH 6.0 myocardial sections placed in a water bath for 30 min for antigen recovery, followed by 15 min of cooling time. Then, they were washed with tap water and phosphate buffered solution (PBS). TBS-T solution with 3% hydrogen peroxide incubation was used to suppress endogenous peroxidase then incubated overnight at 4 °C with anti-nitrotyrosine antibody 1:300 (cat# N0409) (Sigma Aldrich, St. Louis, MO, USA). After washing, biotin-conjugated secondary antibody min and di-amino benzidine and hydrogen peroxide chromogen substrate were added on the slide for 30 min. All slides were stained with hematoxylin and mounted. Tissue sections were analyzed and imaged using a light microscope Nikon E400 (Nikon Instrument Group, Melville, NY, USA) [16].

### 4.4. Measurement of PARP and Caspase 3 Activation

Frozen left ventricular tissues were prepared using a tissue grinder and buffer lysis. The protein concentration was determined with a Bio-Rad protein assay (Bio-Rad Laboratories, Hercules, CA, USA). The lysate samples were separated by electrophoresis on sodium dodecyl sulfate-polyacrylamide gel electrophoresis (SDS-PAGE), nitrocellulose membranes (Amersham Biosciences, Piscataway, NJ, USA), and blocked with 5% nonfat milk in Tris-buffered saline with Tween (TBST) for 2 h at room temperature. Membranes were incubated with an antibody against cleaved caspase-3 1:300 (cat#9661) and cleaved-PARP 1:300 (cat#5625) (Cell Signaling Technology, Danvers, MA, USA), in 5% milk/TBST at 4 °C overnight. After overnight incubation in primary antibody, membranes were washed generously with TBS-Tween 20 solution for 20 min. Washed membranes were incubated with secondary HRP-conjugated antibodies at a dilution of 1:10,000, and HRP-substrate chemiluminescence was used through Syngene Western blotting detection system. The quantification of protein band densities was analyzed by ImageJ 1.37 software as we explained previously [17].

### 4.5. Statistical Analysis

SPSS software version 21 (SPSS Inc., Chicago, IL, USA) was used for analysis of data in all the treatment and control groups. Mean ± SD was calculated for all the measurements. Student’s *t*-test and Mann–Whitney nonparametric tests, were used to compare the two groups. *p*-values less than 0.05 were measured as statistically significant.

## 5. Conclusions

In conclusion, better preservation of high energy phosphates and improved coronary blood flow as well as cardiac function can be achieved with FTY720 pretreatment during heart transplantation. FTY720 also inhibits caspase-3 and PARP-1 signaling pathways that contribute to cardioprotection.

## Figures and Tables

**Table 1:** Baseline parameters of Fingolimod (FTY720) treated group and control group.

Hemodynamic Variables	A1 and C1 (FTY720 Group) Baseline	B1 and D1 (Control Group) Baseline	*p* Value
Aortic systolic pressure (mmHg)	114.4 ± 1.9	116.6 ± 2.8	0.2783
Aortic diastolic pressure (mmHg)	90.6 ± 3.1	93.7 ± 3.7	0.4210
Body weight (g)	319 ± 8.2	327 ± 11.4	0.3136
Heart rate/min	364 ± 7.6	359 ± 11.5	0.5398
Left ventricular end-systolic pressure (mmHg)	116.6 ± 5.1	115.6 ± 4.9	0.4298
Left ventricular end-diastolic pressure (mmHg)	7.6 ± 0.8	7.4 ± 0.5	0.3746
Left ventricular dP/dT_max_ (mmHg/s)	7246 ± 189.2	6987 ± 158.8	0.4330
Left ventricular dP/dT_min_ (mmHg/s)	−9002 ± 387.3	−9180 ± 299.6	0.5673
